# Stroke in Transcatheter Aortic Valve Implantation (TAVI): A Comprehensive Review

**DOI:** 10.3390/jcm14196754

**Published:** 2025-09-24

**Authors:** Dimitrios N. Nikas, Lampros Lakkas, Sotirios Nikopoulos, Konstantinos Tsamis, Xenofon Sakellariou, Matilda Florentin, Panagiotis Papanagiotou, Katerina K. Naka, George Ntaios, Lampros Michalis

**Affiliations:** 1 1st Cardiology Department, Ioannina University Hospital, 45500 Ioannina, Greece; soternikopoulos@gmail.com; 2 Physiology Department, Medical School, Ioannina University, 45500 Ioannina, Greece; l.lakkas@uoi.gr (L.L.); ktsamis@uoi.gr (K.T.); xensakel@gmail.com (X.S.); anaka@cc.uoi.gr (K.K.N.); lamprosmihalis@uoi.gr (L.M.); 3 2nd Internal Medicine Department, Ioannina University Hospital, 45500 Ioannina, Greece; mflorentin@uoi.gr; 4 1st Radiology and Neuroradiology Department, Areteieio Hospital, 11855 Athens, Greece; papanagiotou@me.com; 5 1st Propeduetic Department of Internal Medicine and Stroke Unit, AHEPA University Hospital, 54636 Thessaloniki, Greece; gntaios@auth.gr

**Keywords:** stroke, TAVI, prevention, cerebral protection devices

## Abstract

Transcatheter Aortic Valve Implantation (TAVI) has revolutionized the treatment of severe aortic stenosis in high-risk and inoperable patients. Despite significant advancements in patient selection, techniques, and the evolution of TAVI devices, stroke persists as a consistent adverse event over time, presenting a devastating complication of TAVI procedures and exerting a significant negative prognostic impact. Both acute and subsequent strokes following TAVI continue to pose significant challenges, with substantial implications for patient morbidity and mortality. This paper reviews the incidence, mechanisms, risk factors, and preventive strategies for stroke in TAVI, highlighting recent advancements, particularly in current protective devices, and ongoing challenges in minimizing this adverse outcome.

## 1. Introduction

Severe aortic stenosis is the most common valvular disease in developed countries and is linked to a poor prognosis, with mortality rates reaching approximately 50% within the first two years of symptom onset if left untreated [1]. Transcatheter aortic valve implantation (TAVI) has revolutionized the treatment of severe aortic stenosis, particularly in patients deemed high-risk for surgical aortic valve replacement (SAVR). Since the first percutaneous aortic valve implantation in 2002, TAVI technology has significantly evolved, enabling the treatment of a broader range of patients—from low to very high risk—while reducing complication rates [2,3]. Despite its minimally invasive nature, TAVI carries a risk of stroke, which remains a major complication associated with increased morbidity and mortality [4]. Understanding the incidence and epidemiology of stroke in TAVI is crucial for improving patient outcomes and developing preventive strategies.

## 2. Definition of TAVI-Related Stroke

Neurologic events, including stroke, following TAVI have been identified and measured using a diverse range of clinical, functional, and radiographic methods in both practice and research. Understanding the strengths and limitations of these approaches is essential to evaluating their reliability as surrogate endpoints for patients and their role in clinical trials comparing different management strategies for aortic valve disease. Historically, there has been a lack of consistency in how neurologic events were diagnosed and defined across studies. This inconsistency has sometimes resulted in inaccurate comparisons between contemporary clinical trials and historical literature or surgical databases that were based on differing definitions. A notable example pertains to the incidence of stroke events in patients undergoing surgical aortic valve replacement (SAVR). The 2008 Society of Thoracic Surgeons (STS) report estimated a 1.5% stroke risk with isolated surgical aortic valve replacement; however, these events were classified based on site-reported data, which may not always be reliable [5]. Illustrating this issue, a prospective study found that when strokes were classified based on neurological evaluations conducted before and after surgery, 34 clinical strokes were identified among 196 patients aged 65 and older undergoing SAVR (17.3%), a number much higher than that currently acknowledged. However, only 13 of these 34 cases were ultimately recorded in the STS database [6]. Therefore, apart from the obvious embolic nature of the stroke during TAVI procedures, several other mechanisms, like hypoperfusion or arrhythmia episodes, have to be considered as possible causes of stroke in TAVI.

**Figure 1:**
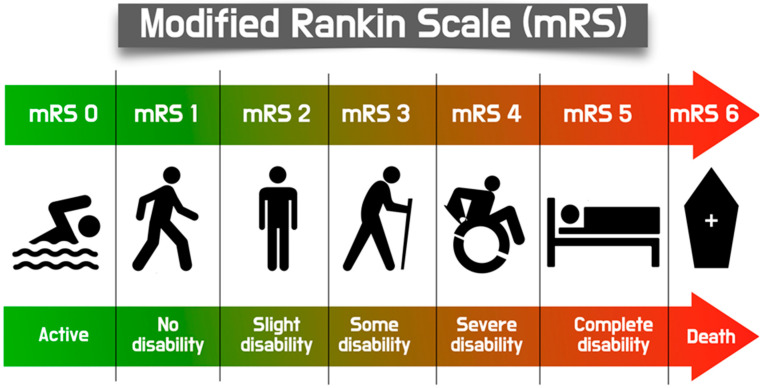
Schematic illustration of the modified Rankin Scale, a commonly used scale to assess the degree of disability or dependence in individuals who have experienced a stroke or other neurological events.

Based on the Valve Academic Research Consortium (VARC-2) criteria, stroke is defined as an acute neurological dysfunction lasting at least 24 h and/or confirmed by imaging evidence of cerebral infarction. It was further classified using the modified Rankin Scale (mRS) (Figure 1) at 30 days as major stroke (score > 2) or minor stroke (score < 2). Additionally, stroke was categorized by timing in relation to TAVI as a procedural event (<24 h), a periprocedural event (subacute stroke 1–30 days), or a late stroke (up to 1 year after TAVI) [7]. Since many patients may present with subclinical cerebral ischemia, one of the most important methods for detecting subclinical microemboli, especially with regard to the exact step of the procedure, is through monitoring of intracranial arterial circulation, using transcranial Doppler (TCD). High-intensity transient signals (HITS) can be recorded and assessed based on their presence, absence, or total count. However, the exact cause of these Doppler signals remains uncertain. HITS have been hypothesized to represent solid microemboli, cavitation, gaseous bubbles, or even artifacts, leading researchers to develop various artifact elimination algorithms for improved accuracy in studies. Today, TCD is a valuable tool to identify the type and source of potential embolism towards the brain, providing substantial help in stroke prevention and identifying patients at high risk for stroke in several endovascular procedures, and TAVI in particular [8,9]. Diffusion-weighted magnetic resonance imaging is a highly sensitive technique for detecting acute ischemia in the brain and has been widely used as both a clinical and research tool to assess neurologic events related to aortic valve procedures [10].

## 3. Incidence

The topic of stroke after TAVI (Transcatheter Aortic Valve Implantation) is an important area of research in cardiovascular medicine. Stroke is one of the key complications that can arise after TAVI, and various studies have been conducted to understand its incidence, risk factors, and prevention strategies. The incidence of stroke after TAVI varies across studies. In order to examine all existing published material and assess the incidence of periprocedural stroke and outcomes in patients undergoing TAVI, Eggebrecht et al. [11] initially developed a meta-analysis. Between January 2004 and November 2011, 53 trials were published, with 10,037 patients receiving transfemoral, transapical, or trans-subclavian TAVI for native aortic valve stenosis. TAVI was associated with an average 30-day stroke/TIA rate of 3.3 ± 1.8% (range 0–6%), according to the study’s findings. The majority of these CVEs were major strokes during the first 30 days and were linked to higher mortality rates [11]. Afterwards, Vlastra et al. [12] analyzed a total of 10,982 patients from the CENTER trial (CVE in Patients Undergoing Transcatheter Aortic Valve Implantation With Balloon-Expandable Valves Versus Self-Expandable Valves), undergoing transfemoral TAVI between 2007 and 2018. Strokes occurred in 261 individuals (2.4%) in the first month following TAVI. The median interval between TAVI and stroke was 24 h. Procedures carried out throughout the early years of TAVI (2007–2012) and those conducted during the more recent years (2013–2018) had similar stroke rates (2.4%; *p* = 1.0) [12]. Between February 2011 and June 2021, Okuno et al. [13] examined the SwissTAVI Registry’s short- and long-term stroke incidence and predictors following TAVI. A total of 11,957 patients were included from the registry. One-tenth (11.8%) of the patients had a history of cerebrovascular event and one-third (32.3%) had a history of atrial fibrillation. The average 30-day incidence rate of stroke was 3.0%, with 69% of stroke events occurring within the first 48 h after TAVI [13]. At one year, the incidence of stroke was 4.3%, and at five years, it was 7.8%. Patients who underwent TAVI had a greater risk of stroke for up to two years following the procedure as compared to an age- and sex-matched population, followed by a comparable risk after 760 days. Almarzooq et al. [14] aimed to assess the association of TAVI-related ischaemic stroke using a federal health insurance program database. All Medicare beneficiaries who performed an initial TAVI operation between January 2012 and December 2017 were enrolled. Using ICD codes, authors defined as TAVI-related stroke every ischemic stroke occurring during the index hospitalization for the TAVI procedure. Among 129,628 primary TAVI patients, 5549 (4.3%) had a procedure-related stroke. Procedure-related ischemic stroke during TAVI remains a critically important and potentially preventable source of patient mortality, morbidity, and healthcare utilization [14]. In summary, while TAVI offers a less invasive alternative to traditional surgical valve replacement, it carries a measurable risk typically between 2% and 5% after the procedure, for both major and minor strokes, particularly in the early postoperative period. However, the risk can vary depending on several factors, including the patient’s individual risk profile. Ongoing research aims to refine strategies to reduce the risk, including the use of cerebral protection devices, improved procedural techniques, and better patient selection.

## 4. Possible Mechanisms for Ischemic Stroke After TAVI

According to the definition of TAVI-related stroke, it can occur as an early (within the first week) or late complication of the TAVI procedure. The timing of stroke onset is closely associated with distinct risk factors, and the majority of TAVI-related strokes have been shown to result from an embolic mechanism. There is discrimination in risk factors depending on the onset of symptoms. Early stroke may occur mainly due to several procedural factors like catheter manipulation in the ascending aorta and aortic arch or during the balloon valvuloplasty. Valve positioning and crossing of a severely calcified aortic valve may also lead to the dislodgment of aortic debris [15,16]. Manipulation of large devices like large-bore sheaths, large valvuloplasty balloons, or the valve-device itself may lead to endothelial damage and subsequent activation of platelets and coagulation cascade, which may lead to thrombus formation and further increase the risk of stroke [17,18]. These findings are corroborated by both morphological and imaging studies. Morphological analyses have identified the embolic material as predominantly composed of calcium, valvular tissue, and collagen fibers. Regardless of their composition, emboli appear to be randomly distributed across one or multiple cerebral territories [19]. Gas embolism, associated with large catheters, device manipulation, and contrast dye injection, has also been suggested as a potential cause of stroke in TAVI procedures. Previous data has identified gas micro-embolization, using transcranial Doppler in patients undergoing TAVI procedures 9 (Table 1).

Hemodynamic deterioration during TAVI procedures may also contribute as a factor for periprocedural stroke. Systemic hypotension, caused either due to prolonged rapid ventricular pacing or any extended hemodynamic compromise, may contribute to cerebral hypoperfusion, particularly in the border zones between different cerebral artery territories [20]. Even though a numerically higher rate of stroke has been observed at 30 days after transfemoral TAVI with balloon-expandable valves compared to self-expanding ones, this difference did not reach statistical significance, indicating that stroke rates are independent of device type [21,22]. Special attention should be given to device preparation and deployment due to rare cases of unfortunate embolization of materials or parts of devices, like distal valvuloplasty balloon covers (balloons-guards) [23].

Apart from the acute periprocedural stroke, several other factors may contribute to the occurrence of late stroke events. New-onset atrial fibrillation (NOAF) post-TAVI is not so uncommon, and therefore, undetected or untreated atrial fibrillation (AF) may be associated with stroke events several months post-procedure [24]. Additionally, the lack of endothelization of the valve frame may become a site of micro-thrombi that could potentially embolize and cause stroke events of varying severity [25,26]. Nevertheless, the general patient’s condition, advanced age, and increased atherogenic burden in the supra-aortic vasculature remain significant factors that could potentially lead to future stroke events. Data from the SwissTAVI Registry, which included 11,957 patients, showed that individuals undergoing TAVI had a higher risk of stroke compared to an age- and sex-matched population for up to two years following the procedure, after which the stroke risk became comparable between the two groups [13].

## 5. Predictors of Stroke After TAVI

Several patient-specific and procedural factors contribute to the risk of stroke, both early and late, in TAVI. Identifying these factors can aid in refining patient selection and optimizing procedural strategies.

One of the most important predictors of early stroke in TAVI patients is the patient’s own native valve and aorta anatomical characteristics. Small aortic valve area and annulus size, baseline high gradient, extensive cusp calcification, and presence of annulus calcification or “porcelain aorta” have all been associated with increased risk for stroke [12,27,28]. Even though there was no evidence to increase the rate of clinical stroke events, increased intima-media thickness (IMT) of the ascending aorta has been shown to be associated with an increased risk of cerebrovascular events (CVE), as detected with routine MRI in a small number of post-TAVI patients [29].

## 6. Pathophysiology and Technical Aspects Behind Stroke Causes (Figure 2)

As previously mentioned, stroke related to TAVI can be categorized into two types: acute strokes that occur during or immediately after the procedure, and subacute or late strokes (more than 48 h after TAVI) associated with the patient’s condition or the prosthesis itself. Several studies have demonstrated that the incidence of stroke following TAVI peaks over the first one to two days (24–48 h), with other studies indicating that nearly half of all strokes occur within the first month. However, long-term strokes have also been observed, potentially due to factors like thrombosis or progression of other cardiovascular disease [4]. The clinical manifestations of these strokes can vary significantly. They may range from subclinical events detected through cerebral imaging studies to cases of transient confusion or acute neurological symptoms. In more severe cases, a large stroke may present with clear clinical symptoms and lead to significant disabilities. Overt stroke presents as one of the most severe and devastating complications of TAVI, closely linked to growing morbidity and mortality. It significantly raises the average 30-day mortality rate, making it more than six times elevated in patients who experience a stroke in opposition to those who do not following TAVI [12]. Despite the advancements in device technology and the implementation of new techniques, the overall rate of stroke has not decreased as expected. In the large US TAVI registry, spanning from 2011 to 2017, the rate of 30-day stroke remained constant. Out of the 101,430 patients, 2.3% experienced a stroke within 30 days, with 48.9% occurring within the first day and 68.4% within three days. Notably, the occurrence of stroke was associated with a significant increase in 30-day mortality. Interestingly, both the dual antiplatelet and the oral anticoagulant therapies administered at discharge failed to reduce the 30-day stroke risk [30].

## 7. Acute Stroke (Case Example)

**Figure 3:**
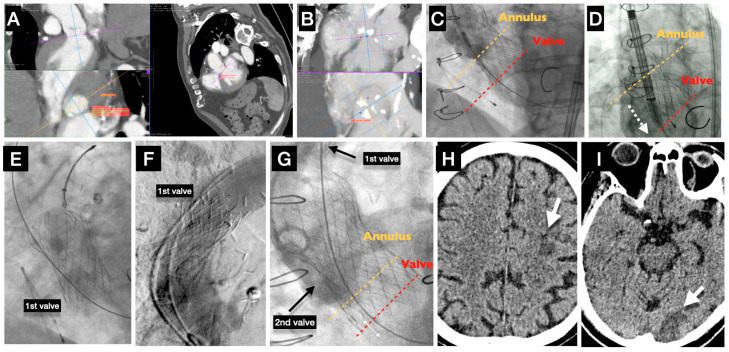
A case example of an acute stroke in a patient who had a rare TAVI complication. (**A**): preprocedural CT evaluation showed a calcified degenerative aortic valve stenosis, suitable for a Medtronic EvolutR, 29 mm. (**B**): distribution of calcium in native valve’s cusps. (**C**): The first valve deployment failed because it migrated to the LV with severe aortic valve regurgitation. (**D**): Attempt to insert a second valve complicated by pushing the first valve almost completely into the LV (dotted arrow). (**E**): The first valve was successfully snared up to the ascending aorta. (**F**): The first valve was retrieved and left at the level of the brachio-cephalic trunk. (**G**): The second valve was successfully implanted at the right position without regurgitation. (**H**,**I**): The next day, the patient complained of blurred vision. Two recent ischemic stroke lesions were identified in brain CT (white arrows).

Most cerebrovascular events after TAVI occur in the periprocedural period, are ischemic in nature, and predominantly embolic in origin, while hemorrhagic strokes account for <5% of cases. TAVI and its transcatheter valve components create a prothrombotic environment in the aortic root [31,32]. The mechanical disruption of atheromatous or calcified tissues during various steps of the TAVI procedure (such as balloon aortic valvuloplasty, catheter and device crossing in the aortic arch, valve implantation, or post-dilation) is likely the primary cause of most periprocedural strokes. These actions inevitably lead to the generation of embolic material. Additionally, suboptimal intraprocedural anticoagulation levels, which can lead to thrombus formation on guidewires and catheters, may play a role in the pathophysiology of strokes during TAVI. Periprocedural air embolisms appeared to be another potential cause of CVEs, particularly when they were connected to delivery systems such as large catheters and contrast injection. A cerebral infarct can occur from systemic hypotension-induced cerebral hypoperfusion, particularly in the presence of coexisting intra- or extra-cranial atheromatous arterial stenosis [33]. Cerebral hypoperfusion may occur during rapid ventricular pacing (both before and after dilatation and valve deployment), leading to a severe decrease in cerebral blood flow. Specifically, in patients with heart failure, may experience persistent hypotension that requires inotropic support, particularly following prolonged rapid ventricular pacing [34]. Furthermore, persistent systemic hypotension during any procedure complication when hemodynamic instability is present (severe acute aortic regurgitation, cardiac tamponade, hemorrhage) may result in irreversible brain injury. The need for a second valve implantation, along with advanced age, has been associated with an increased risk for CVEs [35]. (Case Illustration, Figure 3). On the other hand, even though the center’s high TAVI procedural volume was associated with lower in-hospital outcomes, like mortality, vascular complications, and bleeding, it was not associated with stroke (*p* = 0.14) [36].

Aortic arch and ascending aorta have also been identified as anatomical structures from where embolic debris may dislodge towards the brain and create stroke. The presence of protruding (>3 mm), floating, or ulcerated aortic atheromas has been proven to be independently associated with higher risk periprocedural ischemic stroke post-TAVI. Therefore, careful attention and meticulous study of the preprocedural CT angiography is essential to identify those atheromas and take appropriate measures (valve selection, access site) to reduce the possibility of stroke.

The aortic arch and ascending aorta are important anatomical sources of embolic debris that can dislodge towards the brain and cause stroke. The presence of protruding (>3 mm), mobile, or ulcerated aortic atheromas has been shown to independently increase the risk of periprocedural ischemic stroke after TAVI. Therefore, careful evaluation of preprocedural CT angiography is essential to identify such high-risk atheromas and to guide decisions on valve selection and access route, with the aim of minimizing stroke risk [37].

## 8. Subacute/Late Stroke

It is unlikely that TAVI is related to CVE that occur after 48 h following the procedure. Less is known about the etiology of delayed CVE, which is associated with multifaceted origins. Potential causes of thrombus include the paravalvular gap with the native valve forced against the aortic wall, the valve’s stent before endothelization, and disruption of the calcified native valve with endothelium erosion [38]. Notably, patients with aortic stenosis frequently had spontaneous echo contrast and intracardiac thrombus, which is typically found in the left atrial appendage, indicating a potentially elevated risk for stroke [39]. Additionally, atrial fibrillation (AF), which has been documented in 30% of patients admitted for TAVI may play an important role in the genesis of late stroke. Higher CHA2DS2VASc scores have been shown to be associated with higher CVEs risk irrespective of the presence of AF [40].

New-onset atrial fibrillation (NOAF) is recognized as one of the most significant predictors of both early and long-term cerebrovascular events (CVEs) following transcatheter aortic valve implantation (TAVI). Early studies reported that up to one-third of patients undergoing TAVI developed NOAF, which was associated with a higher incidence of stroke or systemic embolism, although not with increased mortality at 30 days or one-year follow-up [41]. More recent data indicate that NOAF still occurs in approximately 15% of patients and continues to be among the strongest predictors of 30-day CVEs, particularly during the subacute phase after the procedure [42,43,44]. In this large meta-analysis including 179 studies and 241,712 patients, higher surgical risk, transapical access, peripheral artery disease, renal failure, pulmonary hypertension, and severe mitral regurgitation have been identified as strong predictors for NOAF post-TAVI, indicating that comorbid patients may suffer higher rates of NOAF.

Chronic kidney disease (CKD) alone, apart from being a predictor for NOAF, has been shown to be a strong predictive factor for 30-day cardiovascular events (CVEs). Renal disease facilitates chronic inflammation, oxidative stress, and atherosclerosis, which also leads to endothelial dysfunction and vascular calcification. Studies have shown that after adjusting for age and other cardiovascular risk factors, individuals with renal impairment often have an increased risk of stroke, particularly late-onset strokes [45].

Women are more likely to have small aortic valve annuli, increasing their risk factors for cardiovascular events (CVEs). This is because during valve implantation, there can be significant interaction between the prosthetic and native valves, which may create emboli that can lead to stroke events. Between the prosthetic device and native calcified leaflets, thereby heightening the risk of embolic debris generation and subsequent stroke [46].

## 9. Subclinical Leaflet Thrombosis (SLT)

Recently, subclinical leaflet thrombosis (SLT), a relatively unknown phenomenon has been identified an important potential cause for late stroke events in TAVI patients. Subclinical valve thrombosis refers to the presence of thrombus on transcatheter heart valve leaflets, often detected by advanced imaging such as multidetector computed tomography (MDCT), without overt clinical symptoms or hemodynamic compromise. The phenomenon was first described by Makkar et al., when a small series of patients underwent four-dimensional CT scans (4D-CT) of the bioprosthetic valves, both transcatheter and surgical ones, due to cusp/leaflet thrombus formation [47]. Even though in the majority of the cases, thrombus was successfully resolved with anticoagulation, patients with SLT and reduced motion of the involved cusp experienced higher rates of non-major stroke events. Later data, confirmed the presence and the incidence of SLT in larger patients population, proving that transcatheter valves present significantly higher rates of SLT comparing to the surgical ones (13% vs. 4%, *p* = 0.001) [48]. In the same study, patients with SLT presented with higher Aortic valve gradients, and even though the rate of stroke did not differ significantly between those with SLT and without, the reduced leaflet motion was associated with higher rates of transient ischemic attacks (TIA). SLT was completely resolved in all patients (36 of 36) treated with anticoagulation, either with warfarin or NOAC (warfarin 24 [67%]; NOACs 12 [33%]), while it persisted in 20 (91%) of 22 patients who did not receive anticoagulants.

Even though SLT would be logically associated with increased risk for stroke, this has not yet sufficiently proven. A prospective study of 153 patients undergoing TAVI (age: 78.1 ± 6.3 years; female 44%), showed that hypoattenuated leaflet thickening (HALT) a hallmark of SLT, is linked to greater white matter hyperintensity burden on brain MRI at 6 months post-TAVI, suggesting silent brain injury, though it did not increase cognitive decline or mortality over 3.1 years [49]. In another metanalysis, which included 22 studies with a total of 11,567 patients and found that the presence of SLT was not associated with a higher risk of stroke or TIA. Specifically, the odds ratio (OR) for stroke was 1.06 (95% confidence interval [CI]: 0.75 to 1.50; *p* = 0.730), and for TIA, the OR was 1.01 (95% CI: 0.40 to 2.57; *p* = 0.989), which means that while SLT is relatively common post-TAVI, it may not translate into a higher incidence of cerebrovascular events [50].

It is important to recognize that many studies on subclinical leaflet thrombosis (SLT) report cases occurring without overt clinical complications. A large meta-analysis of 25 studies found a median SLT incidence of 6% at a 30-day median follow-up. Notably, the use of intra-annular transcatheter heart valves was associated with a twofold increased risk of SLT compared to supra-annular valves. Furthermore, patients who developed SLT had a 2.6-fold higher risk of stroke or transient ischemic attack (TIA). Another meta-analysis, which differentiated SLT from clinical valve thrombosis (CVT)—defined as symptomatic valve dysfunction with elevated transvalvular gradients, with or without systemic embolic events or need for reintervention—found that patients with CVT experienced significantly higher rates of stroke [51]. Importantly, patients receiving oral anticoagulation (OAC) had markedly lower stroke rates compared to those on dual antiplatelet therapy (DAPT). These findings suggest that OAC may be the most effective therapeutic option for TAVI patients presenting with CVT.

## 10. Pharmaceutical Treatment for Stroke Prevention in TAVI

The TAVI procedure is associated with increased incidence of thromboembolic stroke, making the implementation of effective prevention strategies essential, keeping always in mind the risk of bleeding. Several clinical trials have evaluated the effectiveness of single and dual antiplatelet therapy, unfractionated heparin, direct thrombin inhibitors, and direct oral anticoagulants [52,53,54].

Recent landmark studies have significantly reshaped the clinical approach to antithrombotic therapy in patients undergoing transcatheter aortic valve implantation (TAVI). For patients without an established indication for long-term oral anticoagulation—such as atrial fibrillation—single antiplatelet therapy (SAPT) is now considered the standard of care. The ATLANTIS trial, a multicenter, international, randomized, open-label, superiority study, compared apixaban with standard antiplatelet therapy in 1500 patients following TAVI [55]. Patients were randomized to receive apixaban 5 mg twice daily (or 2.5 mg according to standard dose adjustment criteria) versus standard-of-care antiplatelet therapy. At one-year follow-up, apixaban did not demonstrate superiority over antiplatelet therapy in reducing thromboembolic events. Similarly, the GALILEO trial assessed the efficacy of low-dose rivaroxaban (10 mg once daily) versus antiplatelet therapy in patients without an indication for anticoagulation [53]. The study was terminated early due to a significantly increased risk of death, thromboembolic complications, and major bleeding in the rivaroxaban group compared to standard antiplatelet therapy. Together, these pivotal trials demonstrate that the use of non-vitamin K oral anticoagulants (NOACs) in TAVI patients without any other clear indication for anticoagulation does not confer additional benefit in reducing thromboembolic events and is associated with an elevated risk of bleeding.

With respect to the standard antiplatelet therapy in patients undergoing transcatheter aortic valve implantation (TAVI), evidence strongly supports the use of single antiplatelet therapy (SAPT) over dual antiplatelet therapy (DAPT). The initial results from the ARTE (Aspirin Versus Aspirin + Clopidogrel Following Transcatheter Aortic Valve Implantation) Randomized Clinical Trial demonstrated for the first time that SAPT tended to reduce the incidence of major bleeding events without increasing the risk of thromboembolic events such as myocardial infarction or stroke [52]. These findings were subsequently confirmed in the larger, randomized POPular TAVI trial (Antiplatelet Therapy for Patients Undergoing Transcatheter Aortic-Valve Implantation) [56]. In this trial, the SAPT group exhibited significantly lower rates of both bleeding and the composite outcome of bleeding and thromboembolic events at one year compared to those who received DAPT for three months post-TAVI. Multiple meta-analyses have consistently reinforced these conclusions, showing that SAPT confers a superior safety profile without compromising ischemic protection. Several meta-analyses have validated these results, suggesting that patients who do not require DAPT for any other reason (e.g., recent coronary or peripheral percutaneous intervention with stent) should be treated with SAPT as the first-line therapy in patients undergoing TAVI.

Special attention should be given to patients presenting with either SLT or CVT. In these patients, oral anticoagulation (OAC) with either vitamin K antagonists (VKAs) or NOACs is recommended. Both agents have demonstrated efficacy in treating SLT and reducing the risk of systemic events, including stroke, in patients with CVT. Patients who are taking DAPT and present with SLT should be switched to OAC [57].

Generally, after the TAVI, the recommended treatment remains aspirin for patients with low thrombotic risk, since dual antiplatelet therapy, rivaroxaban and apixaban, failed to reduce the risk of ischemic stroke and increased the risk of bleeding. For patients already in oral anticoagulation before the TAVI procedure due to atrial fibrillation or other indication, the addition of clopidogrel is not recommended because it was shown to increase bleeding risk [54]. Considered all available data, a more conservative antiplatelet treatment appears to be the safer, yet more beneficial approach to TAVI patients who do not require anticoagulation for any other reason.

## 11. Endovascular Interventions in Acute Treatment of Stroke in TAVI Patients

Mechanical thrombectomy (MT) is the standard of care for acute ischemic stroke due to large-vessel occlusion [58]. However, only a small proportion of post-TAVI stroke patients undergo this therapy. In the international ASTRO-TAVI registry (16,615 procedures), 387 patients (2.3%) experienced stroke, yet only 10% (*n* = 39) received endovascular intervention [59]. While neurointervention did not reduce 1-year mortality, it was associated with significantly better functional outcomes at 3 months (OR 2.9; 95% CI 1.2–7.0; *p* = 0.016). The limited uptake of MT likely reflects factors common in the broader stroke population—such as mild clinical presentation, delayed recognition, or distal vessel occlusions [60]. Nonetheless, case reports suggest that in carefully selected TAVI patients, MT can yield favorable outcomes, even without thrombolysis before [61,62]. Consequently, centers performing TAVI procedures must establish protocols to promptly identify, diagnose, and refer their patients with stroke for mechanical thrombectomy (MT), if they meet the current eligibility criteria [63].

## 12. Devices to Reduce Periprocedural Stroke

Stroke represents a significant complication of transcatheter aortic valve implantation (TAVI), impacting both short- and long-term patient outcomes. The majority of stroke events associated with TAVI are of embolic origin and occur during the peri-procedural period. Consequently, the use of a cerebral protection device is a rational strategy to mitigate the risk of embolization by capturing or deflecting debris generated throughout various procedural steps. Similar devices have been employed for several years in other endovascular interventions, such as carotid artery stenting, demonstrating acceptable efficacy in reducing cerebral embolic complications [64] (Table 2).

**Figure 4:**
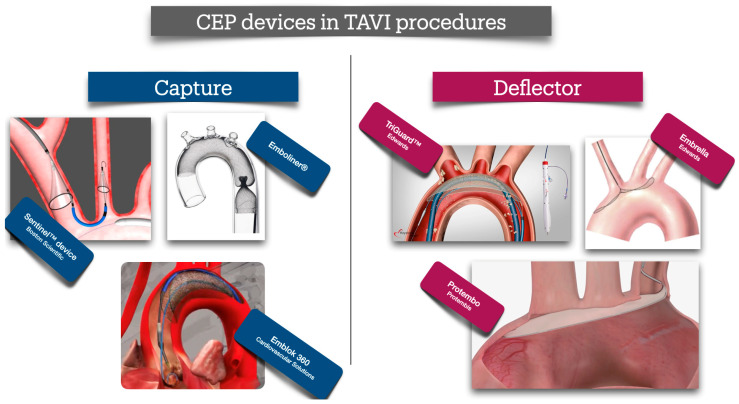
Schematic illustration showing the positioning of major cerebral embolic protection devices during TAVI, separated into two [2] major categories: Caprute and Deflector devices. *Capture devices*: Sentinel™ device deployed via the right radial/brachial artery, with filters in the brachiocephalic and left common carotid arteries. Emboliner^®^ is a whole-aorta capture device. Emblok 360 device is integrated within the femoral sheath, providing full arch and systemic protection. *Deflector devices*: TriGUARD™ is positioned over the aortic arch, deflecting embolic debris away from all three major cerebral vessels. Embrella and ProtEmbo system are similar as both positioned proximally in the aortic arch, using a deflection membrane to shield all supra-aortic vessels. Embrella is positioned through the Right radial access and covers mainly both the carotids, while Protembo is positioned via the Left and offers full supra-aortic branches covering.

Cerebral protection devices for TAVI procedures are broadly classified into two categories: capture devices and deflector devices (Figure 4). Capture devices function by physically catching embolic material, mimicking the design of distal protection filters used in carotid interventions. These systems typically consist of a filter or net-like structure deployed within or proximal to the carotid arteries, preventing embolization to the cerebral circulation. In contrast, deflector devices are typically composed of large mesh-like structures deployed in the aortic arch, designed to cover the three major supra-aortic vessels. These devices permit uninterrupted cerebral perfusion while simultaneously preventing embolic debris from entering the cerebral circulation by redirecting it distally toward the descending aorta.

The most widely utilized cerebral protection device in TAVI procedures is the Sentinel™ Cerebral Protection System (Boston Scientific, Marlborough, MA, USA). This system comprises two independent filters: one positioned within the brachiocephalic trunk (BCT) and the other in the left common carotid artery (LCCA), thereby offering protection to critical regions of the anterior cerebral circulation. The device captures and removes embolic debris released during the procedure, enabling subsequent histopathological analysis of the retrieved material. A key advantage of the Sentinel™ system is its exclusive deployment via the right radial artery, allowing precise positioning of the filters, the first one in the BTK and the second one in LCCA, using a dedicated steering mechanism. Importantly, the device does not interfere with the delivery or positioning of the TAVI valve, ensuring an uncomplicated, easy, and safe procedure. However, in patients with no available radial access—such as those with a history of multiple radial interventions—the deployment of the Sentinel™ system is not feasible, necessitating the selection of an alternative cerebral protection strategy. Another important disadvantage is that the Sentinel™ device cannot completely protect both the anterior and posterior cerebral circulation. While the filters, placed in BCT and the LCCA, can prevent embolization in both the anterior and the right posterior circulation, the system cannot protect the left posterior circulation, leaving those cerebral areas vulnerable to potential embolism. Even though the larger stroke events occur mainly in the anterior circulation, ischemic lesions may also occur in a significant percentage of TAVI patients in the posterior circulation, especially in those presenting with calcified AoS compared to those with non-calcified AoS (48.8% vs. 14.7%, *p* < 0.001) [65]. This could probably explain the findings of the PROTECTED TAVI trial, in which the routine application of the Sentinel™ device did not reduce the overall incidence of stroke events, but only the large, disabling ones [66].

Initial observational data from large-scale, well-documented registries suggested a modest but borderline-significant reduction in stroke rates with cerebral embolic protection (CEP). Analysis of the STS/ACC TVT Registry, which included 414,649 TAVI patients between 2018 and 2023—of whom 53,389 (12.9%) received a SENTINEL device—demonstrated a small yet statistically significant reduction in stroke incidence [67]. Patients with a prior history of stroke were identified as being at increased risk for disabling stroke and appeared to derive greater benefit from CEP compared to other subgroups.

These findings formed the rationale for subsequent large-scale randomized controlled trials designed to evaluate the true clinical efficacy of routine CEP use. The PROTECTED TAVI trial was a landmark, prospective, multicenter, randomized, controlled trial conducted to test the efficacy of the routine use of the Sentinel™ device in patients undergoing transfemoral TAVI. The study examined whether cerebral embolic protection (CEP) devices could reduce the risk of periprocedural stroke. Three thousand (3000) patients were included in the study, with half of them assigned to CEP. The study concluded that the routine use of CEP during TAVI procedures failed to reduce the overall risk of stroke. However, the results do not rule out the usefulness and potential benefit of CEP use, especially in patients at high risk for stroke, like those with previous CVE, a highly calcified aortic valve, or renal failure [12]. Moreover, despite the higher number of female patients in the CEP group, the Sentinel™ device reduced the “large” disabling strokes, making a valuable impact on the outcome of the patients; even though not completely proven, it appears that female TAVI patients are known to experience a higher rate of periprocedural stroke compared to male ones [30].

The same results were also confirmed by the British Heart Foundation (BHF) PROTECT-TAVI trial. This was a large-scale, multicenter, randomized controlled study designed to evaluate the efficacy of routine cerebral embolic protection (CEP) during transcatheter aortic valve implantation (TAVI) in reducing the incidence of stroke [68]. Being the largest so far trial of its kind, it represented a vast majority of the everyday TAVI procedures performed in the UK (almost 30% of all TAVIs in the UK), enrolling 7635 participants across 32 National Health Service hospitals and one private practice in the UK. The trial aimed to provide definitive evidence on the clinical efficacy and cost-effectiveness of using routine CEP with the Sentinel™ device. The trial reported relatively lower numbers of successful CEP device deployment (81.2%) and stopped prematurely due to futility. The trial concluded that routine use of CEP during TAVI did not reduce the risk of stroke, leading to questions about the utility of CEP devices in this setting. The overall rate of stroke occurred in 81 of 3795 participants (2.1%) in the CEP group and in 82 of 3799 participants (2.2%) in the control group (difference, −0.02 percentage points; 95% confidence interval, –0.68 to 0.63; *p* = 0.94). Contrary to the original PROTECTED TAVI trial, the routine use of CEP in this study did not reduce the rate of disabling stroke, raising concerns regarding the real clinical need for CEP devices during TAVI procedures. Critics, however, noted that the study was not sufficiently powered to detect differences in disabling stroke rates and disabling stroke was included in the secondary endpoints of the study. None of the pre-specified groups have shown significant benefit in terms of stroke reduction. Therefore, additional data and larger trials may be required to identify which patient sub-groups are at a higher risk of stroke and the actual benefit in disabling stroke reduction.

In the same principle of capturing debris during TAVI procedures, a different whole-body protection device has been designed. The Emblok 360° Whole Body Embolic Protection, Innovative Cardiovascular Solutions, Grand Rapids, Michigan, has been designed to capture and remove embolic material, protecting patients during TAVI procedures from distal embolism from both cerebral and systemic circulation without impeding blood flow. The basic concept of the device regards the deployment of a single large net-filter in the ascending aorta, proximal to the BCT, and parallel to the TAVI device via the contralateral femoral route. This proximal position allows sufficient capturing and removal of all potential embolic material created at the site of valve placement during the procedure. In the First-In-Man study conducted to evaluate the feasibility of complete cerebral protection during TAVI procedures, the Emblok device demonstrated easy and successful deployment with uncomplicated retrieval in all cases without any neurological events [69]. Even though a remarkable position time of 4.2 min (±5.4 min) was shown, the total new lesion volume, as estimated with Magnetic Resonance Imaging (MRI) of the brain, was not different from other trials testing CEB devices. Thus, for the time being, no conclusive results regarding the efficacy of the Emblok device can be drawn.

The Emboliner^®^ Embolic Protection Catheter (Santa Cruz, CA, USA) is a novel “full-body” embolic protection device. It features a cylindrical Nitinol mesh filter that conforms circumferentially to a wide range of aortic anatomies, while its expandable access port is compatible with various procedural devices. A key advantage of the Emboliner^®^ is its utilization of the secondary arterial access typically used for pigtail catheter insertion during transcatheter aortic valve implantation (TAVI), thereby eliminating the need for additional vascular access. The device is deployed from just proximal to the brachiocephalic trunk (BCT) down to the descending aorta, effectively covering and protecting all three supra-aortic vessels. This positioning enables the capture of embolic debris as it migrates toward the descending aorta, preventing distal embolization to critical vascular territories such as the renal and mesenteric arteries. Moreover, unlike deflector-type devices, the Emboliner^®^ not only captures but also facilitates the retrieval of embolic material, allowing for post-procedural analysis and pathological examination of the captured debris. Results from the SAFE-PASS 2 study showed 100% implantation success rate and 30-day major adverse cardiac and cerebrovascular events (MACCE) of 6.5% [70]. Since the device deflects and collects the debris, significant conclusions can be drawn from the materials it collects. Histopathology revealed predominantly acute thrombus and valve or arterial tissue, with smaller amounts of calcified tissue. Notably, self-expanding valves were associated with twice the number of particles exceeding 150 µm (*p* = 0.0281).

Apart from capture devices, several other devices, designed to reduce periprocedural stroke during TAVI, have also evolved. Generally, most of the rest of the devices have been designed to mainly “deflate”, rather than “capture”, debris away from the supra-aortic vessels. The ProtEmbo^®^, Protembis GmbH, Aachen, Germany, is a net-like device, designed to cover all three supra-aortic arteries and deflate embolic particles away from the cerebral and towards the peripheral circulation. This novel low-profile device is deployed via the left radial access, using a 6Fr-guiding sheath, positioned in the proximal to the BTC. The PROTEMBO C Trial was an international, multi-center, single-arm trial evaluating the safety and feasibility of the ProtEmbo (Protembis GmbH) CEP system, compared to historical controls (non-inferiority) [71]. Thirty-seven of 41 enrolled patients underwent TAVI with the ProtEmbo^®^ device where both primary endpoints were achieved, with low 30-day MACCE (8.1%) and high technical success (94.6%); DW-MRI findings showed reduced lesion volumes compared to historical controls, and only one stroke occurred in a patient whose device was removed prematurely. The study was interrupted due to the COVID-19 pandemic, and the final report was published two years later, which confirmed the results of the initial report [72]. However, no control, randomized data are yet available.

Another deflector device, placed via the right radial or brachial access, is the Embrella Embolic Deflector System, Edwards Lifesciences, Irvine, CA, USA. It consists of a Nitinol shaft and frame, which includes heparin-coated polyurethane membrane with 100-μm-sized pores. The device is designed to cover the ostia of the brachiocephalic trunk (and its right carotid branch) and the left carotid artery, originating directly from the transverse aorta, thereby deflecting emboli away from the cerebral circulation. Initial reports in four [4] patients demonstrated easy placement, and safe deployment of the device [73]. A larger study in 52 patients, confirmed the safety and feasibility of the device, without however preventing the occurrence of cerebral microemboli, but it was associated with a reduction in lesion volume as measured with cerebral DW-MRI post TAVI [74]. In another study, 15 patients undergone TAVI using the Embrella device; the number, size and volume of the new DW-MRI brain lesions, were compared to 37 patients who had previously undergone TAVI without a protection device. The Embrella device increased the number of newly detected brain lesions, which however, accompanied by a significant reduction in single-lesion volume and the absence of large total infarct volumes. The majority of the Embrella patients’ lesions were found in the right hemisphere, whereas the comparable group had lesions in both hemispheres, raising concerns regarding the origin of the lesions and their association with the access site and deployment technique [75]. Again, no randomized data to prove the actual benefit of the device are still available.

At the same concept lies the main principle of the TriGuard™ 3 deflector device. The TriGuard™ 3 (Keystone Heart, Tampa, FL, USA) is consisting of a nitinol frame and a dome-shaped mesh deflector, delivered transfemorally via the contralateral to the primary femoral access site. It is designed to “self-position” in the aortic arch, proximally to the BCT, allowing the TriGuard™ 3 to conform to a variety of patient anatomies. The initial studies (the DEFLECT I and III trials), performed to evaluate the early versions of the device (TriGuard™ and TriGuard™ HDH), have shown reductions in new ischaemic brain lesions and total lesion volume (TLV) per patient in diffusion-weighted magnetic resonance imaging (DW-MRI) [76,77]. In the DEFLECT I trial, 37 consecutive patients underwent TAVI procedures with the use of TriGuard™. The presence of new cerebral ischaemic lesions on post-procedure DW-MRI (*n* = 28) was similar to historical controls (82% vs. 76%, *p* = NS). However, an exploratory analysis found that per-patient total lesion volume was 34% lower than reported historical data (0.2 vs. 0.3 cm^3^), and 89% lower in patients with complete (*n* = 17) versus incomplete (*n* = 10) cerebral vessel coverage (0.05 vs. 0.45 cm^3^, *p* = 0.016) [76]. In the DEFLECT III trial, 87 patients were randomized to undergo TAVI procedures with or without protection with the TriGuard™ device; complete cerebral vessel coverage was achieved in 89% of subjects (per treatment group) [77]. In those per-treated patients, TriGuard™ HDH use was associated with greater freedom from new ischaemic brain lesions (26.9 vs. 11.5%), fewer new neurologic deficits detected by the National Institutes of Health Stroke Scale (3.1 vs. 15.4%), and improved neuro-cognitive function at 30 days. These two early trials established TriGuard™ as an effective and safe protection implementation to avoid potential cerebral embolic events during TAVI procedures.

The REFLECT I trial was a larger, single-blinded, randomized study that enrolled 375 patients (including 54 roll-in and 204 randomized participants) undergoing transcatheter aortic valve implantation (TAVI) with or without cerebral protection using the TriGuard™ device for [78]. The study met its primary safety endpoint, demonstrating a significantly lower 30-day early safety event rate compared to the predefined performance goal (21.8% vs. 35%, *p* < 0.0001). However, the primary efficacy endpoint was not achieved, although there was a numerical reduction in covert cerebral embolic events in the TriGuard™ arm compared to controls.

Being the largest clinical evaluation of the TriGuard™ device to date, REFLECT I confirmed its safety profile but failed to demonstrate consistent efficacy in preventing cerebral embolic injury. A key limitation was the incomplete coverage of all three supra-aortic vessels, achieved in only 57.3% of patients, mainly due to device displacement, passage of the TAVII delivery system behind the filter, entanglement between the TriGuard™ and TAVI systems, or premature withdrawal of the protection device.

These findings highlight a common limitation among current cerebral embolic protection devices: the challenge of achieving complete and stable cerebral coverage throughout the procedure. Additional data coming from recent metanalysis of studies looking at the efficacy of CEP devices have been inconclusive, urging the need for further randomized data in order to finally clarify their clinical benefit [79,80]. Notably, the larger British Heart Foundation (BHF) PROTECT-TAVI trial which evaluated the Sentinel™ device, had not yet been published, and therefore not included at the time of the metanalysis. By the time this review was written, the results from the BHF study had just been published, and once more, they replicated the results of the PROTECT-TAVI trial, showing no significant difference in the primary endpoint of any stroke within 72 h or before discharge.

The BHF PROTECT-TAVI trial was designed to be a larger, more definitive study than PROTECTED TAVR, and its results suggest that the routine use of the Sentinel device does not offer a significant benefit in preventing strokes during TAVR [68]. In this large trial, funded by the British Heart Foundation, investigators randomized 7635 patients (mean age, 81 years; 39% women) to undergo TAVR with the Sentinel CEP device or with no CEP device. Approximately 81% of the device group had the device fully deployed in both the right innominate and left carotid arteries. The primary endpoint, which was any stroke within 72 h after TAVR or before hospital discharge (whichever occurred first), showed similar rates in both the device group and the no-device group (2.1% vs. 2.2%). Similarly, the secondary endpoints of disabling stroke (1.2% vs. 1.4%) and death within 8 weeks (2.1% vs. 1.9%) also exhibited similar rates. The results were generally consistent across the prespecified subgroups and remained consistent even after accounting for the completeness of device deployment.

In general, despite the rationale and the initial encouraging results, for the use of cerebral embolic protection (CEP) devices in transcatheter aortic valve implantation (TAVI), current evidence remains inconclusive regarding their consistent clinical benefit in preventing periprocedural stroke. While devices like the Sentinel™ have demonstrated safety and feasibility, their efficacy appears limited primarily to reducing large, disabling strokes—without significantly impacting the overall incidence of stroke. Deflector systems such as TriGuard™ and ProtEmbo^®^, and whole-body protection platforms like Emblok, have introduced novel approaches to cerebral protection, yet most have been evaluated in small cohorts or early-phase studies, with inconsistent efficacy outcomes and a lack of randomized data. A key limitation across existing CEP technologies lies in the inability to achieve complete and stable coverage of all supra-aortic vessels, often due to anatomical variability, procedural challenges, or device limitations. Furthermore, the heterogeneity in stroke mechanisms, lesion distribution, and patient-specific risk profiles complicates efforts to demonstrate universal benefit from CEP use. A comprehensive overview of the literature supporting the efficacy and safety of the CEP devices is presented in Table 3.

Future research should prioritize large-scale, adequately powered randomized controlled trials with rigorous neurologic and neuroimaging endpoints to determine which patient populations derive the greatest benefit. Special attention should be given to high-risk subgroups—such as patients with prior stroke, severe aortic calcification, or chronic kidney disease—as well as sex-specific differences in periprocedural stroke incidence. Additionally, technological refinements aimed at enhancing device stability, deployment success, and comprehensive vessel coverage are essential. Ultimately, a personalized, risk-based approach to cerebral protection may be the key to maximizing neurologic safety in contemporary TAVI practice.

In the near future, artificial intelligence (AI) is expected to play a significant role in enhancing cerebral embolic protection (CEP) strategies during TAVI procedures, as it has already done in other high-risk stroke diseases [81,82]. AI-driven models could improve risk stratification algorithms by identifying patients at elevated risk, thereby guiding the selective use of CEP devices. These models would likely integrate both clinical and anatomical data—such as the presence of aortic arch calcification or atheromatous plaques—derived from preprocedural imaging work-up. Moreover, AI could facilitate the appropriate selection and accurate deployment of CEP devices by analyzing the same datasets. This would ensure complete coverage of the supra-aortic vessels and maximize embolic capture or deflection efficacy, depending on the type of protection device utilized.

## 13. Conclusions

Stroke remains a significant complication during transcatheter aortic valve implantation (TAVI), with a substantial impact on both patient prognosis and family outcomes. Although recent advancements in TAVI devices and procedural techniques have contributed to a reduction in stroke incidence, these improvements have not been sufficient to eliminate the risk. Consequently, further strategies are required to enhance patient protection. Future developments in embolic protection devices are expected to overcome current limitations by improving device stability, ensuring comprehensive cerebral and systemic coverage, and incorporating artificial intelligence for real-time procedural optimization. Nevertheless, technological innovation alone will be insufficient; meticulous patient selection will be critical to maximizing the clinical benefits of these next-generation devices. Identifying patients at highest risk for embolic events and tailoring protection strategies accordingly will be essential to translating these innovations into meaningful improvements in stroke prevention and overall TAVI outcomes.

## Figures and Tables

**Figure 2:**
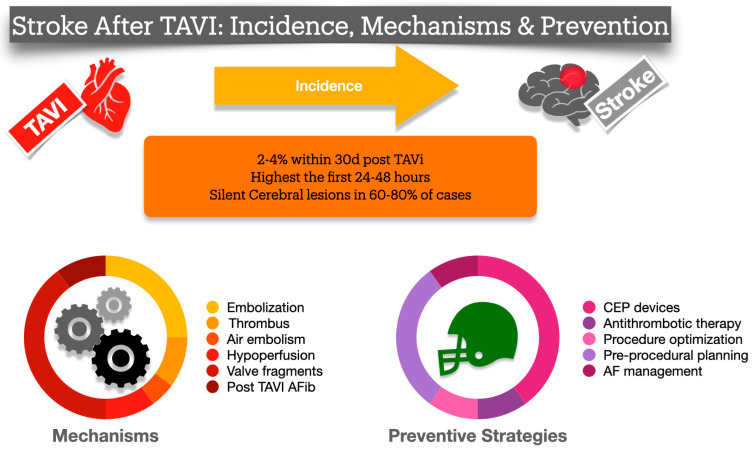
Comprehensive illustration of the basic information regarding incidence, mechanisms and prevention strategies in Stroke during or after TAVI procedures.

**Table 1:** Possible Mechanisms for ischemic stroke after TAVI.

**Early Stroke**
Procedural factors Endothelial damage and the activation of platelets and the coagulation cascade activation during the procedure Gas embolism Peri-procedural atrial fibrillation Systemic hypotension
**Late Stroke**
6. Post-procedural atrial fibrillation 7. Lack (total or partial) of endothelization of the stent material of the valve 8. Denudation of endothelium leading to disruption of the native aortic valve 9. General atheromatic/atherothrombotic burden of the patient

**Table 2:** Basic Technical Characteristics of available Cerebral Protection Devices.

Device Name	Manufacturer	Type	Deployment Access	Cerebral Coverage	Key Characteristics
Sentinel CPS	Boston Scientific, St. Paul, MN, USA	Filter	Right radial artery (6 Fr sheath)	Brachiocephalic and left carotid arteries (partial; ~90% cerebral flow, excludes left vertebral artery)	Dual-filter system; captures debris; FDA-approved (2017); high technical success (94.5%); no significant stroke reduction in PROTECTED TAVR trial (RR 0.88, *p* = 0.566).
TriGuard 3	Keystone Heart, Caesarea, Israel	Deflector	Femoral artery (9 Fr sheath)	All major cerebral arteries (innominate, left carotid, left subclavian)	Nitinol mesh (130-µm pores); deflects debris to descending aorta; improved cognitive outcomes in DEFLECT III; ongoing REFLECT trial (NCT02536196).
Embrella	Edwards Lifesciences, Irvine, CA, US	Deflector	Right radial artery (6 Fr sheath)	Brachiocephalic and left carotid arteries	Polyurethane membrane; deflects debris; higher lesion rates on MRI in some studies; not widely adopted.
Emboliner	Emboline, Inc., Santa Cruz, CA, USA	Filter	Femoral artery (9 Fr sheath)	Full cerebral (all supra-aortic branches) and non-cerebral vessels	Cylindrical nitinol mesh; captures 5× more debris (>150 µm) than Sentinel in SafePass 2; TAVR-focused, no CAS data; investigational.
ProtEmbo	Protembis GmbH, Aachen, Germany	Deflector	Left radial artery (6 Fr sheath)	All major cerebral arteries	Low-profile heparin-coated mesh; deflects debris; ongoing PROTEMBO C trial (NCT04205916); investigational.

**Table 3:** Supportive literature regarding safety and efficacy of available cerebral protection devices for TAVI procedures.

Device	Study Design	Basic Results	Clinical Meaning
Sentinel CPS	Randomized controlled trial (*n* = 363 TAVI patients)	94.5% technical success; reduced new cerebral lesion volume on MRI (*p* = 0.03); no significant stroke reduction at 30 days (5.6% vs. 9.1%, *p* = 0.25); debris captured in 99% of filters.	Reduced only the large strokes. Currently no indication for routine usage
TriGuard 3	Randomized controlled trial (*n* = 258 TAVI patients)	85.7% technical success; improved cognitive outcomes (*p* = 0.04); reduced total lesion volume on MRI (34% less, *p* = 0.057); no significant stroke reduction (8.3% vs. 11.4%, *p* = 0.48).	Unproven clinical benefit for stroke reduction. No indication for routine usage.
Embrella	Prospective pilot study (*n* = 52 TAVI patients)	97.5% technical success; debris deflected in all cases; higher new lesion rates on MRI vs. controls (*p* = 0.02); no stroke reduction (10% vs. 11%, *p* = 0.89); limited adoption.	No randomized data. Decreased the size, yet increasing the number of lesions. Limit the new large lesions. No clinical benefit. No evidence for beneficial clinical use.
Emboliner	Prospective, non-randomized study (*n* = 24 TAVI patients)	100% technical success; debris captured in 100% of cases (5× more particles >150 µm than Sentinel); no stroke rates reported; investigational, promising for full cerebral protection.	Easy to deploy, efficient to capture debris. No evidence for clinical application.
ProtEmbo	Prospective feasibility study (*n* = 30 TAVI patients)	100% technical success; debris deflected in all cases; reduced lesion volume on MRI (*p* = 0.06); no stroke rates reported; ongoing PROTEMBO C trial	Successful deployment. Reduced lesion size. No clinical data on reducing clinically evident stroke complication

## Data Availability

No new data were created or analyzed in this study.
